# miR-143-null Is against Diet-Induced Obesity by Promoting BAT Thermogenesis and Inhibiting WAT Adipogenesis

**DOI:** 10.3390/ijms232113058

**Published:** 2022-10-27

**Authors:** Jie Liu, Jiatao Liu, Dewei Zeng, Huan Wang, Yun Wang, Jiali Xiong, Xingping Chen, Junyi Luo, Ting Chen, Qianyun Xi, Qingyan Jiang, Yongliang Zhang

**Affiliations:** 1 Guangdong Provincial Key Laboratory of Animal Nutrition Control, College of Animal Science, South China Agricultural University, Guangzhou 510642, China; 2 Jiangxi Province Key Laboratory of Animal Nutrition, College of Animal Science and Technology, Jiangxi Agricultural University, Nanchang 330045, China

**Keywords:** obesity, BAT, WAT, miR-143, thermogenesis, adipogenesis

## Abstract

Excessive energy intake is the main cause of obesity, and stimulation of brown adipose tissue (BAT) thermogenesis has emerged as an attractive tool for anti-obesity. Although miR-143 has been reported to promote white adipocyte differentiation, its role in BAT remains unclear. In our study, we found that during HFD-induced obesity, the expression of miR-143 in BAT was significantly reduced, and the expression of miR-143 in WAT first increased and then decreased. Knockout (KO) of miR-143 with CRISPR/Cas9 did not affect the energy metabolism of normal diet fed mice and brown adipocyte differentiation but inhibited the differentiation of white adipocytes. Importantly, during high fat diet-induced obesity, miR-143KO significantly reduced body weight, and improved energy expenditure, insulin sensitivity, and glucose tolerance. Further exploration showed that miR-143KO reduced the weight of adipose tissue, promoted mitochondrial number and functions, induced thermogenesis and lipolysis of BAT, increased lipolysis, and inhibited lipogenesis of white adipose tissue (WAT). Our study considerably improves our collective understanding of the function of miR-143 in adipose tissue and its potential significance in anti-obesity and provides a new avenue for the management of obesity through the inhibition of miR-143 in BAT and WAT.

## 1. Introduction

Obesity and its related disorders have become a global pandemic. In September 2021, United Nations Secretary-General Guterres pointed out at the United Nations Food Systems Summit that 2 billion people around the world are overweight or obese, i.e., ∼30% of the world population. Many studies have shown that long-term obesity can also cause other metabolic-related diseases, such as type 2 diabetes [1,2,3]. Obesity arises from a long-term imbalance of energy, with energy intake exceeding the storage capacity of adipose tissue, which leads to energy “overflowing” to ectopic sites.

It is well-established that adipose tissue is essential for controlling energy homeostasis, and adipose tissue can be broadly divided into brown adipose tissue (BAT) and white adipose tissue (WAT) according to its coloration rather than location [4,5]. In recent decades, BAT has been widely concerned because of its unique thermogenic function [6,7]. The strategies of activating and expanding BAT increase energy expenditure in animal models provide a therapeutic promise to anti-obesity [6,8]. WAT, which represents more than 95% of the adipose mass [9], stores excess energy in the form of triglycerides (TGs), and increased accumulation of WAT is a key factor of obesity [10]. Inhibiting lipid storage and promoting lipid mobilization in WAT are also important strategies to prevent obesity.

It is well-known that microRNAs (miRNAs) are linked to obesity and energy metabolism [11,12], and enhancing the thermogenic function of adipose tissue through miRNAs is a promising method for anti-obesity [9,13]. As a member of the miR-143/145 cluster, miR-143-3p is the main expression form of miR-143. Previous studies [14,15] have established that miR-143 is highly expressed in BAT and WAT, compared with its expression in other tissues. So far, a large number of studies have shown that miR-143 promotes white adipocyte differentiation by targeting mitogen-activated protein kinase kinase 5 (MEK5) and extracellular signal-regulated kinase 5 (ERK5) [16,17,18,19,20,21,22]. However, the effect of obesity of miR-143 on adipose tissue remains controversial. Some studies suggest that obesity could reduce the expression level of miR-143 in WAT of mice [15,17,23,24], and other studies have reported that the expression of miR-143 in WAT is positively related to the body weight of mice or adipocyte volume in swine [21,25,26,27]. The role of miR-143 during obesity remains obscure, and still needs further investigation. More importantly, our published data show that cold exposure stimulates stronger body temperature in miR-143 knockout (KO) mice, indicating that miR-143 may be involved in cold-induced thermogenesis [28]. However, the function and mechanisms of miR-143 in BAT are still unclear.

Therefore, the purpose of this paper is to explore the effect of miR-143 in obesity and the role of miR-143 in BAT and WAT in obese mice. Our study firstly analyzed the miR-143 expression pattern in BAT and WAT of mice induced by a high-fat diet (HFD). Then, CRISPR/Cas9 technology was used to construct whole-genome miR-143KO mice. We examined the phenotypes of WT and KO mice fed normal diet (ND) and HFD, respectively. Our study found that miR-143KO did not affect the energy metabolism of mice fed with ND. However, it can significantly promote the energy expenditure of mice induced by HFD. We further show that miR-143KO could significantly promote thermogenesis in BAT and inhibit adipogenesis in WAT, which contributes to anti-obesity.

## 2. Results

### 2.1. miR-143 Is Downregulated in BAT of Mice Induced by HFD

**Figure 1:**
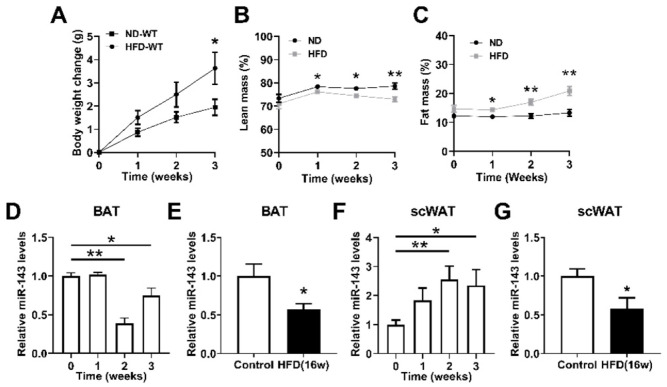
miR-143 is downregulated in BAT of mice induced by HFD. (**A**) The body weight of mice fed HFD or ND (n = 8). (**B**,**C**) The fat and lean mass of mice fed ND or HFD (n = 8). (**D**,**F**) The miR-143 expression in BAT (**D**) and scWAT (**F**) of mice fed HFD for indicated times lengths (n = 8). (**E**,**G**) The miR-143 expression in BAT (**E**) and scWAT (**G**) of mice fed HFD for 16 weeks (n = 8). Data are presented as the mean ± SEM. * *p* < 0.05 vs. controls; ** *p* < 0.01 vs. controls, as determined by a two-tailed unpaired Student’s *t*-test.

We first analyzed the changes of miR-143 in BAT and subcutaneous white adipose tissue (scWAT) after HFD induction. The percentage of fat mass increased and the percentage of lean mass decreased after feeding for one week, while increased body weight was observed (Figure 1A) three weeks later (Figure 1B,C). The expression of miR-143 in BAT was significantly reduced two weeks after HFD feeding, and this reduction kept on up to 16 weeks (Figure 1D,E). On the other hand, the expression of miR-143 in scWAT increased significantly in 2 and 3 weeks, but decreased in 16 weeks (Figure 1F,G). These results suggested that the expression of miR-143 is in a different profile in BAT and scWAT, indicating its diverse roles.

### 2.2. miR-143KO Inhibits the Differentiation of White Adipocytes, but Not Brown Adipocytes

**Figure 2:**
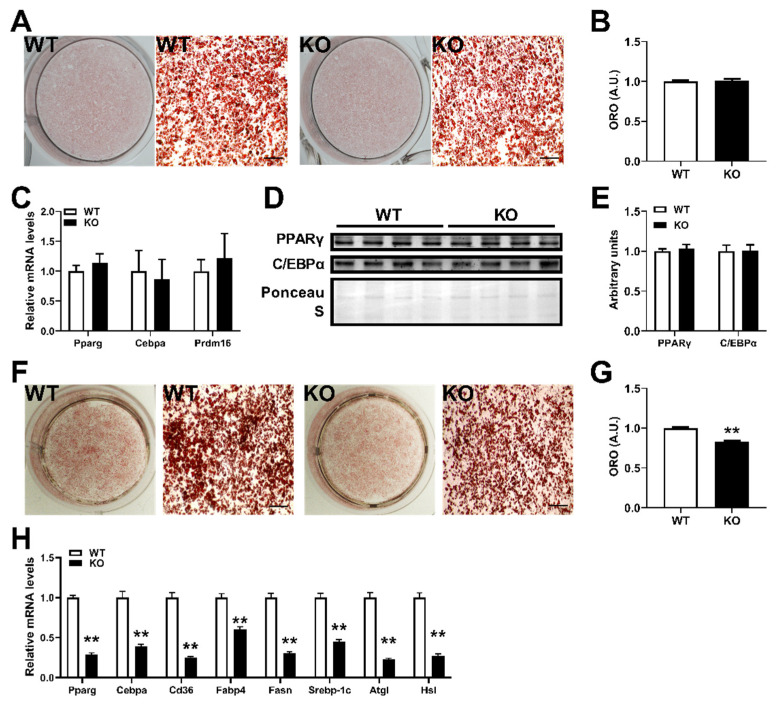
miR-143KO inhibits the differentiation of white adipocytes, but not brown adipocytes. (**A**,**B**) The Oil-Red-O staining of WT or KO brown adipocyte lipid droplets (**A**) and quantified by measuring absorbance post-isopropanol extraction of Oil Red O (**B**) (n = 3). (**C**–**E**) The adipogenic-related genes mRNA (**C**) (n = 6) and PPARγ and C/EBPα protein (**D**,**E**) (n = 4) expression levels in the differentiated WT or KO brown adipocyte. (**F**,**G**) Oil red O staining of white adipocytes and statistical analysis of oil red O after isopropanol extraction (n = 3). (**H**) Lipogenesis- and lipolysis-related genes mRNA levels (n = 8). ** compared with the control group, *p* < 0.01.

To explore the effect of miR-143 on the differentiation of brown and white adipocytes, we harvested the stromal-vascular fraction (SVF) from the BAT and WAT, differentiated this cell population ex vivo into mature adipocytes, and found no significant difference in oil red O staining between WT and KO brown adipocytes (Figure 2A,B). Moreover, peroxisome proliferator-activated receptor (PPAR)-γ and CCAAT enhancer-binding protein (CEBP)-α were also not significantly altered (Figure 2C–E). On the contrary, miR-143KO inhibited white adipocyte differentiation (Figure 2F), and the expression of adipogenesis and lipogenesis genes (Figure 2G,H). These results further indicate the diverse role of miR-143 in brown and white adipocytes during differentiation.

### 2.3. miR-143KO Does Not Affect Energy Metabolism in Mice

**Figure 3:**
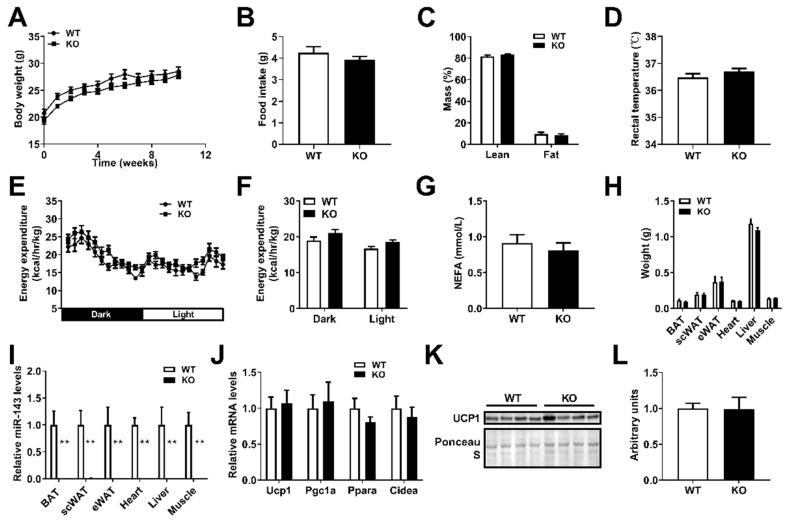
miR-143KO does not affect the phenotype related to energy metabolism in mice. (**A**) The body weight of WT and KO mice fed ND (n = 10). (**B**–**D**) Food intake (**B**), fat mass (**C**), and rectal temperature (**D**) of WT and KO mice fed ND (n = 10). (**E**,**F**) The energy expenditure of 8-week-old WT and KO mice fed ND for 10 weeks (n = 4). (**G**) The NEFA levels in the serum of 8-week-old WT and KO mice fed ND for 10 weeks. (**H**) The organ weights of WT and KO mice (n = 10). (**I**) The miR-143 expression in adipose tissue, heart, liver, and muscle (n = 8). (**J**–**L**) The thermogenic-related genes mRNA expression levels (n = 8) and UCP1 protein expression levels in the BAT of WT and KO mice fed ND. Data are presented as the mean ± SEM. ** *p* < 0.01 vs. controls. Two-tailed unpaired Student’s *t*-test.

In order to evaluate the potential contributions of miR-143 to the development of obesity, CRISPR/Cas9 technology was used to construct a global miR-143 KO mouse [29]. However, miR-143KO showed neither phenotypic abnormalities nor energy imbalance, and changes in BAT thermogenesis in mice fed a ND under normal conditions (Figure 3A–L). Thus, under a normal diet and housing conditions, miR-143KO did not lead to any phenotype changes on energy metabolism in mice.

### 2.4. miR-143KO Reduces Diet-Induced Obesity by Promoting Energy Expenditure

**Figure 4:**
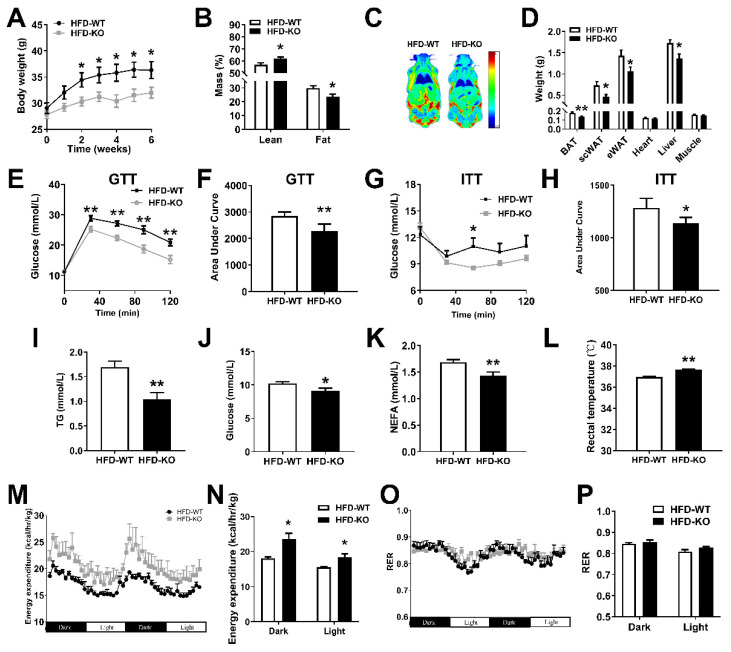
miR-143KO reduced HFD-induced obesity by increasing energy expenditures in BAT. (**A**–**D**) Body weight of WT and KO mice fed HFD (n = 8). (**B**–**D**) fat mass (**B**), quantitative magnetic resonance (**C**), and organ weight (**D**) in WT and KO mice fed HFD for 16 weeks (n = 8). (**E**–**H**) The glucose tolerance test (n = 12) and insulin tolerance test (n = 8) of mice fed HFD for 10 days. (**I**–**K**) The TG, glucose, and NEFA levels of serum of mice fed HFD for 16 weeks (n = 8). (**L**) The rectal temperature of WT and KO mice fed HFD for 16 weeks (n = 8). (**M**–**P**) Energy expenditure and RER of 8-week-old WT and KO mice fed HFD for 10 days (n = 4). Data are presented as the mean ± SEM. * *p* < 0.05 vs. controls; ** *p* < 0.01 vs. controls, as determined by a two-tailed unpaired Student’s *t*-test.

Since miR-143KO did not lead to any phenotype changes in mice under a normal diet, HFD was used to induce obesity in KO mice. Interestingly, the body weight of KO mice was significantly lower than that of WT mice (Figure 4A). The reduced body weight was primarily due to a significant fat mass reduction (Figure 4B,C), and the weight of adipose tissues and liver of KO mice were significantly lower than those of WT mice (Figure 4D). In addition, the tolerance tests were performed, showing an improvement in insulin sensitivity and glucose tolerance in miR-143KO mice (Figure 4E–H). It is conclusively considered that circulating triglycerides (TGs) are negatively correlated with obesity and insulin resistance [30], and circulating non-esterified fatty acids (NEFAs) are negatively correlated with energy expenditure [31]. We also observed that the concentrations of circulating TGs, glucose, and NEFAs were decreased in KO mice (Figure 4I–K). More importantly, the rectal temperature of KO mice was significantly higher than that of WT mice (Figure 4L) and the energy expenditure increased significantly at the early (Figure 4M,N) and final stages (Figure S1A,B) of HFD feeding. The respiratory exchange ratio (RER) remained unchanged, which may be due to the simultaneous increase of glucose and lipid utilization (Figure 4G,H and Figure S1C,D). Thus, our results showed that miR-143KO reduced diet-induced obesity by increasing energy expenditure.

### 2.5. miR-143KO Promotes Thermogenesis and Lipolysis in BAT of Mice Fed with HFD

**Figure 5:**
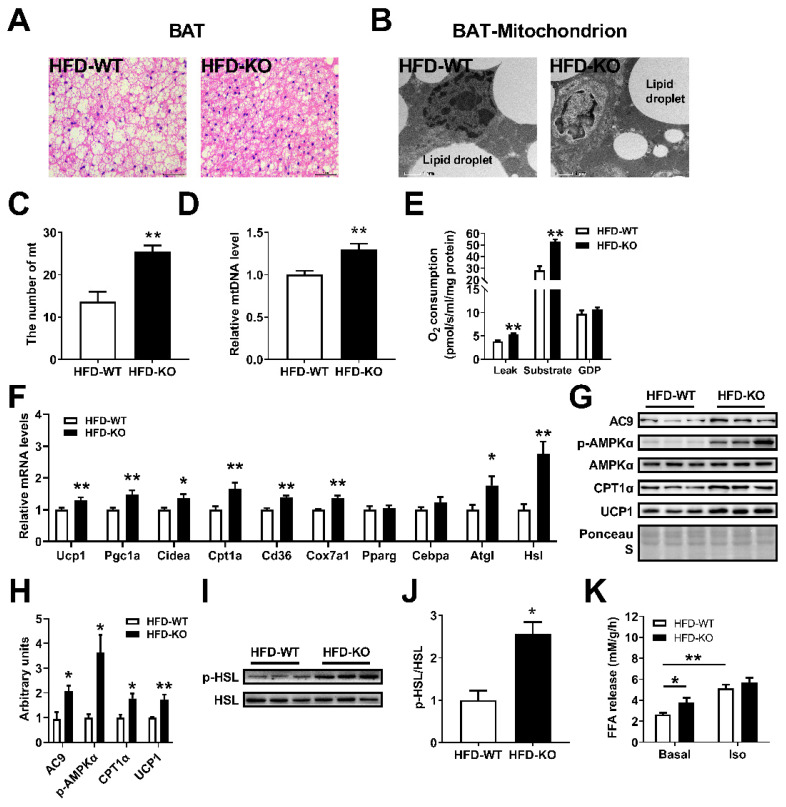
miR-143KO promoted thermogenesis and lipolysis of BAT in HFD fed mice. (**A**,**B**) Morphologic analysis of adipocytes and mitochondria in BAT of WT and KO mice. Scale bars: 100 μm and 1 μm. (**C**,**D**) Mitochondria number and the relative copy number of mitochondrial DNA in BAT of WT and KO mice (n = 8). (**E**) The oxygen consumption of mitochondria isolated from BAT of WT and KO mice fed HFD for 1 week (n = 4). (**F**–**J**) The thermogenic and lipolysis-related genes mRNA (n = 8) and protein expression levels in the BAT of WT and KO mice fed HFD. (**K**) Fatty acid release capacity of BAT from WT and KO mice fed HFD for 1 week (n = 4). Data are presented as the mean ± SEM. * *p* < 0.05 vs. controls; ** *p* < 0.01 vs. controls, as determined by a two-tailed unpaired Student’s *t*-test.

The thermogenic function of BAT is of great significance to anti-obesity. Mitochondrion is the basis of brown adipocytes thermogenesis. We found decreased adipocyte sizes (Figure 5A) and a higher number of mitochondria in BAT of KO mice fed with HFD (Figure 5B–D). Then, BAT mitochondria were isolated and mitochondrial respiration [32] was significantly increased at basal and substrate-induced states in KO mice, and this increase was inhibited by guanosine diphosphate (Figure 5E). Hormone-sensitive lipase (HSL) is the key enzyme of lipolysis [33]. Moreover, the expression levels of thermogenic and lipolysis-related genes and p-HSL proteins were increased in BAT of KO mice, while adipogenic mRNAs such as PPAR-γ and CEBP-α were not significantly altered (Figure 5F–J). Correspondingly, a lower accumulation of lipid droplets was observed in KO mice (Figure 5A). In addition, compared with BAT from WT mice, BAT from KO mice exhibited stronger NEFAs release capacity under basal condition, but no significant difference under isoproterenol stimulation (Figure 5K). These data suggested that the enhancement of thermogenesis in BAT is an important aspect for anti-obesity of miR-143KO.

### 2.6. miR-143KO Promotes Lipolysis and Inhibits Adipogenesis in WAT of Mice Fed with HFD

**Figure 6:**
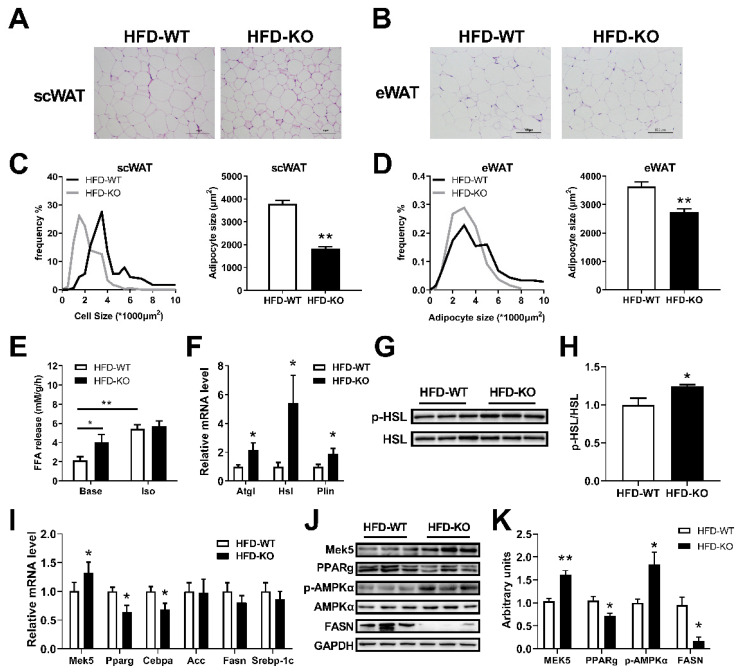
miR-143KO promoted lipolysis and inhibited the adipogenesis of WAT in HFD-fed mice. (**A**,**B**) Morphologic analysis of adipocytes in scWAT and eWAT. Scale bars: 100 μm. (**C**,**D**) The adipocyte sizes and the frequencies of the cell size were calculated. Three sections from different individuals (n = 3) per group were randomly selected. (**E**) Fatty acid release capacity of scWAT in WT and KO mice fed HFD for 1 week (n = 4). (**F**–**H**) The lipolysis-related genes mRNA (n = 8) and protein expression levels in the scWAT. (**I**–**K**) The adipogenic-related genes mRNA (n = 8) and protein expression levels in the scWAT. Data are presented as the mean ± SEM. * *p* < 0.05 vs. controls; ** *p* < 0.01 vs. controls, as determined by a two-tailed unpaired Student’s *t*-test.

We further studied the effect of miR-143KO on lipolysis and adipogenesis in WAT of mice fed with HFD. MiR-143KO significantly reduces the area and distribution of scWAT and epididymal adipose tissue (eWAT) (Figure 6A–D). The scWAT of KO mice showed a stronger release capacity of NEFAs under basal condition (Figure 6E), while the lipolysis-related genes and p-HSL protein were increased as well (Figure 6F–H). Moreover, miR-143KO significantly increased the mRNA (Figure 6I) and protein (Figure 6J,K) levels of MEK5, which is one of the target genes of miR-143 [18]. Furthermore, miR-143KO significantly decreased adipogenic-related genes, PPARγ and FASN protein and increased p-AMPKα, protein in scWAT (Figure 6I–K). Thus, the increased lipolysis and inhibited adipogenesis in WAT could contribute to the suppression of obesity by miR-143KO.

## 3. Discussion

Various miRNAs are associated with obesity [34] and obesity-related metabolic diseases [35,36]. Among them, miR-143 is closely related to the differentiation and adipogenesis of precursor adipocytes [16,17,18,19,20,21,22], and it is deemed as a novel regulator of type 2 diabetes [15,36]. However, the effect of miR-143 on the thermogenic function of BAT has not been reported. In the present study, the expression of miR-143 was found to decrease significantly in BAT of mice induced by HFD (2, 3 and 16 weeks), suggesting a potential regulatory role of miR-143 to BAT function in obesity. Despite the fact that miR-143 promotes white adipocyte differentiation and adipogenesis [16,17,18,19,20,21,22], reports on the effect of obesity on the expression of miR-143 in WAT are still inconsistent [15,17,21,23,24,25,26,27]. Interestingly, our study found that the expression of miR-143 was increased in scWAT at the early stage of HFD feeding but decreased in long-term HFD feeding (16 weeks), and this bidirectional change needs further exploration. Besides, adipose tissue contributes significantly to the circulating miRNA pool (about 66% of circulating exosomal miRNA) [37], and circulating miR-143 and obesity are intimately linked [23,38,39,40,41], which further indicated the involvement of miR-143 in adipose tissue in the regulation of obesity.

To further study the function of miR-143 in BAT and WAT, CRISPR/Cas9 technology was used to construct whole-genome miR-143KO mice, which harvested the SVF cells from the BAT and WAT and differentiated this cell population ex vivo into mature adipocytes. It was found that miR-143KO did not affect the differentiation of brown adipocytes, but significantly inhibited the differentiation of white adipocytes, which was consistent with previous studies on the differentiation of white adipocytes by miR-143 [16,17,18]. These results suggest that miR-143 plays different roles in brown and white adipocytes. Importantly, we did not detect any major phenotypic abnormalities between WT and KO mice under ND and housing conditions, including body weight, food intake, rectal temperature, energy expenditure, the weight of tissues, and BAT function. Previous studies have shown that genomic deletion of miR-143/145 will not lead to obvious developmental defects, and the overexpression of miR-143 in BAT and liver also does not affect the development of mice [42,43,44]. Notably, our published data show that cold exposure stimulates stronger body temperature in miR-143KO mice [28], indicating that miR-143 may be involved in cold-induced thermogenesis. Therefore, we speculate that miR-143 in adipose tissue acts as a stress miRNA upon metabolic challenge just like miR-21 [45]. Thus, we evaluated the role of miR-143 in an HFD-induced obesity model. Compared with WT mice, the increase in body weight and fat mass of KO mice was significantly decreased during HFD feeding. In addition, improvements in insulin sensitivity and glucose tolerance were observed in miR-143KO mice, which further support the anti-obesity of miR-143KO. Another group has reported that that miR-143 impairs glucose metabolism [15], since the improvement of impaired glucose and lipid metabolism are common events in anti-obesity experiments [46]. The above results indicate that miR-143KO inhibits diet-induced obesity.

Activating BAT thermogenesis is an important anti-obesity strategy and obesity can lead to impairment of BAT thermogenesis [6,8]. We also found energy expenditure significantly increased in miR-143KO mice fed with HFD, since rectal temperature and energy expenditure were both increased, and circulating TGs, glucose, and NEFAs were all decreased, indicating consumption of thermogenic materials. Non-shivering thermogenesis (NST) is mediated by UCP1 located in mitochondria, and uses protons generated through an electron transport system to generate heat, rather than ATP synthesis [47]. Our research found that miR-143KO significantly promoted thermogenesis by increasing protein expression of AC9 and UCP1 in BAT upon HFD feeding, which further confirmed our data in publishing that miR-143 directly targets AC9 to inhibit BAT thermogenesis, as demonstrated by bioinformatic analysis, dual-luciferase reporter assays, and the AC9 protein analysis of KO mice and overexpression of miR-143 both in WT and KO brown adipocytes. Increasing evidence points toward AMP-activated protein kinase (AMPK) as a specific regulator of various aspects of mitochondrial biology and energy homeostasis [48,49,50]. MiR-143KO increases the protein expression of phosphorylated AMPKα in BAT, indicating that miR-143 may regulate energy metabolism and mitochondrial biology through AC9 and AMPK signaling pathway. We also found that miR-143KO significantly improved the lipolysis of BAT, thereby providing materials for thermogenesis. The above results show that the enhancement of thermogenesis in BAT is the important reason for anti-obesity of miR-143KO.

WAT stores excess energy, and the increased accumulation of WAT is a key factor of obesity [10]. Previously, miR-143 was found to be closely positively related to the differentiation and adipogenesis of precursor adipocytes through MEK5-PPARγ signaling pathway [16,17,18,19,20,21,22]. Our results have shown that the weight of WAT and the size of adipocytes significantly decreased in miR-143KO mice fed HFD. Moreover, miR-143KO significantly inhibited WAT adipogenesis by inhibiting MEK5-PPARγ signaling pathway, which further verified previous studies [16,17,18,19,20,21,22]. It is noteworthy that we observed an 80–90% reduction in FASN protein and an increase in p-AMPKα protein in miR-143KO WAT after HFD induction, whereas *Fasn* mRNA expression showed no difference. The results suggested that miR-143 may strongly inhibit FASN expression at the post-transcriptional level, mainly through AMPK signaling pathway [48,49,50]. More importantly, we found that increased lipolysis of WAT by miR-143KO promoted the expression of WAT-enriched genes and activities HSL protein. Further studies are required to better understand the regulatory mechanism of HSL. Thus, increased lipolysis and inhibited adipogenesis by miR-143KO in WAT could contribute to the suppression of obesity. 

Our study has provided new insights on the function of miR-143 in obesity management. Notably, we newly found that miR-143 played a regulatory role in adipose tissue lipolysis and BAT thermogenesis and provided a theoretical basis for further clinical application of the miR-143. Further investigation using miR-143 adipose tissue-specific knockout mice should give us a more detailed picture on the regulatory roles of miR-143 in adipose tissue function and obesity.

## 4. Materials and Methods

### 4.1. Experimental Animals

MiR-143 KO mice have been previously reported [29]. Experiments were performed using homozygous miR-143KO mice and homozygous WT mice bred by miR-143 knockout heterozygotes. During the ND feeding experiment, WT and KO male mice were divided into two groups according to their body weight at weaning age (ND-WT/KO). During diet-induced obesity studies, eight-week-old WT and KO male mice were divided into two groups according to their body weight (HFD-WT/KO) and fed a 60% kcal HFD (D12492; Research Diet). Mice were housed in a 12-h light–dark cycle in controlled temperature (22 °C–24 °C) and humidity (50–65%) conditions with free access to food and water. Tissues and serum were collected after 6 h of food removal at the end of each experiment, quickly snap-frozen in liquid nitrogen, and then stored at −80 °C for further experiments. All of the experimental protocols and methods were approved by the College of Animal Science, South China Agricultural University (Ethical code number: SCAU-AEC-2015–0527). All of the experiments were conducted following the “The Instructive Notions with Respect to Caring for Laboratory Animals” issued by the Ministry of Science and Technology of the People’s Republic of China.

### 4.2. QMR Analysis of Whole-Body Composition

The body composition of mice was determined using quantitative magnetic resonance (QMR, Niumag Corporation, Shanghai, China) according to the manufacturer’s protocol.

### 4.3. Oil Red O Staining

The primary brown adipocytes were washed with PBS once and fixed with 4% formaldehyde in PBS for 30 min at room temperature and washed thrice with PBS. Then, the cells were stained with Oil Red O (#O1391; Sigma-Aldrich, St. Louis, MO, USA) for 15 min. Following 3 washes with water, lipid droplets were observed and photographed under a microscope (TE2000-E; Nikon, Tokyo, Japan). 

### 4.4. H&E Staining

BAT samples were fixed overnight with 10% formalin and then stored in 50% ethanol. The fixed BAT pads were then embedded with paraffin, sectioned, and stained with hematoxylin and eosin (HE). Pictures of stained BAT tissue were obtained using a Leica Dm6000 M Microscope (Leica, Wetzlar, Germany).

### 4.5. Metabolic Cage Analysis

Animals were first acclimated to the system for 24 h, and measurements of O_2_ consumption, CO_2_ production, and energy expenditure were performed over the next 48 h by CLAMS (Promethion, Sable Systems International, Las Vegas, NV, USA). Food and water were available ad libitum, and RER was calculated by the ratio of the oxygen consumed (VO_2_) relative to the carbon dioxide produced (VCO_2_).

### 4.6. GTT and ITT

For GTT, mice were fasted for 12 h and then injected with glucose (1.5 g/kg) intraperitoneally. For ITT, the mice were fasted for 4 h and then injected with insulin (0.5 U/kg) intraperitoneally. A drop of blood was taken from the tail vein before and after (30 min, 60 min, 90 min, and 120 min) injections of glucose or insulin for the determination of blood glucose with a glucometer. The total area under the glucose concentration curve was calculated using GraphPad Prism software 9.0.

### 4.7. Mitochondrial Observation by TEM

The observation of mitochondria in BAT was performed using transmission electron microscopy according to the routine procedure [51]. In brief, BAT was surgically removed from mice, and then was immediately cut into small pieces of 1 mm^2^, and then fixed in 2.5% glucose taraldehyde. After 24 h of fixation, the tissue samples were post-fixed in 1% osmium tetroxide solution (pH 7.4) and processed into epoxy resin. Ultra-thin sections were cut and stained with uranyl acetate and lead citrate and examined under a transmission electron microscope (TEM; JEOL JEM2100).

### 4.8. Mitochondrial DNA Quantification

The relative amount of nuclear DNA and mitochondrial DNA (mtDNA) was determined by quantitative real-time PCR [52]. The ratio of mtDNA to nucleic DNA reflects the mitochondrial content in a cell. BAT was homogenized and digested with protein K overnight in a lysis buffer for DNA extraction using a DNeasy kit (Qiagen). Quantitative PCR was performed using corresponding primers (Table 1). Real-time PCR was carried out on a STRATAGENE Mx3005P sequence detection system with SYBR Green Master Mix (Promega). The program is 20 min at 95 °C, followed by 50–60 cycles of 15 s at 95 °C, 20 s at 58 °C, and 20 s at 72 °C. mtDNA content was normalized to nuclear DNA content.

### 4.9. BAT Mitochondrial Respiration Assays

Mitochondrial respiration rates were measured using an O_2_K high-resolution respirometer (Oroboros Instruments, Innsbruck, Austria) [32]. Freshly isolated BAT was placed in a pre-cold mitochondrial isolation buffer (250 mM sucrose, 5 mM Tris, and 2 mM EGTA, adjusted to pH 7.4) and homogenized until the tissue was completely lysed. The samples were transferred to centrifuge tubes and centrifuged at 8000× *g* for 10 min. The supernatant was discarded and then resuspended mitochondria in respiration buffer (110 mM mannitol, 0.5 mM EGTA, 3 mM 1 MgCl_2_, 20 mM taurine, 10 mM KH_2_PO_4_, 60 mM K lactobionate, 0.3 mM DTT, and 0.1% BSA (fatty acid free), adjusted to pH 7.1), followed by passive filtration (100 μM). Oxygen consumption was normalized to mitochondrial protein concentration. After stabilization, real-time oxygen consumption was then measured continuously (DatLab software 4.3, Oroboros Instruments, Innsbruck, Austria). Baseline respiration following the addition of substrates (palmatoylcarnitine (4 mM), pyruvate (10 mM), and malate (5 mM)) was then measured. UCP1-dependent respiration was assessed by the addition of 2 mM guanosine diphosphate (HY-113066A, MedChemExpress).

### 4.10. Measurement of Basal and Stimulated Lipolysis of BAT and WAT

The method refers to the previous article [53]. Briefly, BAT or scWAT was surgically removed, weighted, and washed in DPBS. Tissues were cut into small pieces and preincubated in 200 mL DMEM containing 2% BSA (FA-free), 10 mM forskolin/isoproterenol, and 5 mM triacsin C (to inhibit acyl-CoA synthetases) in 96-well plates at 37 °C, 5% CO_2_, and 95% humidified atmosphere for 60 min. For the measurement of basal lipolysis, transfer fat explants into 200 mL of identical, fresh medium and incubated for a further 60 min at 37 °C, 5% CO_2_, and 95% humidified atmosphere. NEFA content was measured in the incubation media using NEFA kit and free glycerol reagent and appropriate standard solutions, respectively. Lipolysis of adipose tissue organ cultures was calculated as nmol FA per mg/tissue.

### 4.11. Isolation and Culture of Primary Brown and White Adipocytes

BAT from 3 to 5-day-old pups and WAT from 21 to 24-day old mice were obtained, and then were minced and digested in PBS containing collagenase type I (1 mg/mL, 17018029, Gibco) and CaCl_2_ (3 mM) at 37 °C for 30 min. The tissue suspension was filtered using 100 μm and 40 μm cell strainers (BD Biosciences), and cell pellets were resuspended in DMEM/F12 GlutaMAX (Invitrogen), supplemented with 10% fetal bovine serum (FBS) and 1% penicillin/streptomycin, and then plated. After 2 days of fusion, SVF cells from BAT were induced to differentiate with medium containing 10% FBS, 1 μM rosiglitazone (R2408, Sigma), 1 μM dexamethasone (D4902, Sigma), 5 μg/mL insulin (I0546; Sigma), 0.5 mM isobutylmethylxanthine (I7018, Sigma), 125 nM indomethacin (I7378, Sigma), and 1 nM T_3_ (T2877, Sigma) for 2 days. The white SVF cells induction medium did not contain T_3_ and indomethacin. Next, the cells were maintained in a culture medium containing 10% FBS, 5 μg/mL insulin, and 1 nM T_3_ (not for white adipocytes). The media was changed every 2 days. Cells were harvested on days 6 or 8 of differentiation for further analysis.

### 4.12. Analysis of TG, Glucose, and NEFA Concentrations

TG, glucose, and NEFA concentrations were assayed using a TG (A110-1-1), glucose (A154-1-1), or NEFA assay kit (A042-2-1) (Nanjing Jiancheng Biotechnology Institute, Nanjing, Jiangsu, China) according to the manufacturer’s protocol.

### 4.13. qPCR Analyses

Total mRNA was extracted using Trizol Reagent (15596-026, Thermo Fisher Scientific, Shanghai, China). After DNase I digestion (2270A, Takara Bio, Kusatsu, Shiga, Japan), a total of 1 μg of total RNA was reverse-transcribed into cDNA using MLV Reverse Transcriptase (M1705, Promega, Madison, WI, USA) and oligo (dT) 18 primer or a specific stem-loop primer for miR-143/145 (3806, Takara Bio). Real-time PCR was carried out in a STRATAGENE Mx3005P sequence detection system with SYBR Green Master Mix (Promega). Results were normalized to the expression of the housekeeping genes Gapdh, 18 s or U6 using the 2^−ΔΔCt^ method. The primer sequences used are presented in Table 1.

### 4.14. Western Blot Analysis

Tissues and cells were lysed in RIPA buffer containing 1 mmol/L PMSF protease inhibitor (P7626, Sigma). The protein concentrations were then measured using a BCA Protein Assay kit (Thermo Fisher Scientific, 23227). Primary antibodies against UCP1 (#ab10983, Abcam, 1:2000), AC9 (ab191423, Abcam, 1:1000), phospho-AMPKα (Thr172, #2535, CST, 1:1000), total-AMPKα (#5831, CST, 1:1000), phospho-HSL (Ser563, #4139, CST, 1:1000), HSL (#4107, CST, 1:1000), CPT1α (#12252, CST, 1:1000), MEK5 (#40737, CST, 1:1000), PPARγ (#2443, CST, 1:1000), and FASN (#3189, CST, 1:1000) were used according to the manufacturer’s instructions. Primary antibodies were incubated in a blocking buffer at 4 °C overnight. Secondary Alexa antibodies from Life Technologies were then added for 1 h. Detection was performed by chemiluminescence (ImmobilonTM Western, Millipore Corporation, Billerica, MA, USA), captured using a FUJI LAS 1000-plus chemiluminescence imaging system. The protein density was quantified and analyzed using Image J 1.52a software.

### 4.15. Statistical Analyses

All of the results were expressed as the mean ± SEM, and analyses were performed using Excel. A Student’s *t*-test was used for a single variable comparison between two groups, and a value of *p* < 0.05 was considered to be statistically significant.

## 5. Conclusions

In conclusion, this article directly confirmed the key role of miR-143 in diet-induced obesity. MiR-143KO can alleviate HFD-induced obesity by increasing thermogenesis of BAT and inhibiting adipogenesis of WAT. Our study considerably improves our collective understanding of the function of miR-143 in adipose tissue and its potential significance in anti-obesity and provides a new avenue for the management of obesity through the inhibition of miR-143 in BAT and WAT.

## Figures and Tables

**Table 1:** Primer sequences for quantitative real-time PCR.

Gene	Forward (5′–3′)	Reverse (5′–3′)
mu-Ucp1	ACTGCCACACCTCCAGTCATT	CTTTGCCTCACTCAGGATTGG
mu-Pgc1a	AGCCGTGACCACTGACAACGAG	GCTGCATGGTTCTGAGTGCTAAG
mu-Cidea	ATCACAACTGGCCTGGTTACG	TACTACCCGGTGTCCATTTCT
mu-Cd36	ATGGGCTGTGATCGGAACTG	TTTGCCACGTCATCTGGGTTT
mu-Cpt1a	CTCCGCCTGAGCCATGAAG	CACCAGTGATGATGCCATTCT
mu-Cox7a1	CCGACAATGACCTCCCAGTA	TGTTTGTCCAAGTCCTCCAA
mu-Pparg	CTGCATCTCCACCTTATTAT	CACAGACTCGGCACTCA
mu-Cebpa	AGAAGTCGGTGGACAAGAACA	TTTGGCTTTATCTCGGCTCT
mu-Atgl	AAAGGACCTGATGACCACC	GCAGCCACTCCAACAAGC
mu-Hsl	AGACACCAGCCAACGGATAC	GCTGGCACGGAAGAAGATAC
mu-Plin	CCATGACGACCAGACAGACAC	CCCAGGTCACTGCGGAGAT
mu-Mek5	AAGCAGCCCAAGGAGAGAC	GAACTGCACGATGAATGGGTG
mu-Fasn	GCTGCGGAAACTTCAGGAAAT	AGAGACGTGTCACTCCTGGACTT
mu-Acc	TGTACAAGCAGTGTGGGCTGGCT	CCACATGGCCTGGCTTGGAGGG
mu-Srebp1c	AACCAGAAGCTCAAGCAGGA	TCATGCCCTCCATAGACACA
mu-U6	CTCGCTTCGGCAGCACA	AACGCTTCACGAATTTGCGT
mu-18s	CTTAGTTGGTGGAGCGATTT	GCTGAACGCCACTTGTCC
Mu-Gapdh	GGAAAGCTGTGGCGTGAT	AAGGTGGAAGAATGGGAGTT
mtDNA-specific PCR	CCGCAAGGGAAAGATGAAAGA	TCGTTTGGTTTCGGGGTTTC
DNA-specific PCR	-GCCAGCCTCTCCTGATGT	GGGAACACAAAAGACCTCTTCTGG
mu-qmiR-143-3p	GGGTGAGATGAAGCACTG	CAGTGCGTGTCGTGGAGT
mu-miR143-3p-RT	GTCGTATCCAGTGCGTGTCGTGGAGTCGGCAATTGCACTGGATACGACGAGCTA	

## Data Availability

The datasets [GENERATED/ANALYZED] for this study can be found in the [jianguoyun] [https://www.jianguoyun.com/p/DSVaFM8Qx9P1ChixjNYEIAA], accessed on 17 October 2022.

## Supplementary Materials

The following supporting information can be downloaded at: https://www.mdpi.com/article/10.3390/ijms232113058/s1.

## Abbreviations

miRNAs	microRNAs
KO	knockout
BAT	brown adipose tissue
WAT	white adipose tissue
scWAT	subcutaneous white adipose tissue
eWAT	epididymal white adipose tissue
TGs	triglycerides
NEFA	non-esterified fatty acids
MEK5	mitogen-activated protein kinase kinase 5
ERK5	extracellular signal-regulated kinase 5
ND	normal diet
HFD	high-fat diet
SVF	stromal-vascular fraction
PPAR-γ	peroxisome proliferator-activated receptor-γ
CEBP-α	CCAAT enhancer-binding protein-α
RER	respiratory exchange ratio
QMR	quantitative magnetic resonance
AC9	adenylate cyclase 9
UCP1	uncoupling protein 1
AMPKα	AMP activated protein kinase α
CPT1α	carnitine palmitoyltransferase 1α
FASN	fatty acid synthase
HSL	hormone sensitive lipase
NST	non-shivering thermogenesis
