# Supplementary material for: miR-143-null Is against Diet-Induced Obesity by Promoting BAT Thermogenesis and Inhibiting WAT Adipogenesis
Source: Int J Mol Sci. 2022 Oct 27;23(21):13058. doi: 10.3390/ijms232113058 (PMC9658130; doi:10.3390/ijms232113058)
Supplement: Supplementary file 1 [file ijms-23-13058-s001.zip › ijms-1944027-supplementary.pdf]

International Journal of
Molecular Sciences

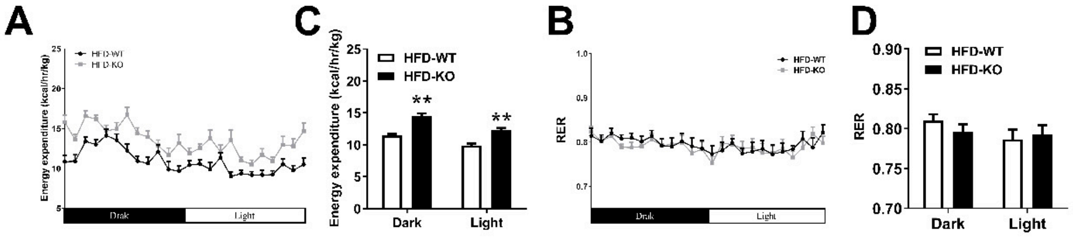

Figure S1. The energy expenditures of WT and KO mice fed HFD for 16 weeks. (A–D) Energy expenditure and RER of 8-week-old WT and KO mice fed HFD for 16 weeks (n = 4). Data are presented as the mean ± SEM. * $P < 0.05$ vs controls; ** $P < 0.01$ vs controls, as determined by a two-tailed unpaired Student's t-test.
